# Severe COVID-19-associated myocarditis with cardiogenic shock – management with assist devices – a case report & review

**DOI:** 10.1186/s12871-022-01890-4

**Published:** 2022-12-12

**Authors:** Stephanie Noone, Armin N. Flinspach, Stephan Fichtlscherer, Kai Zacharowski, Michael Sonntagbauer, Florian J. Raimann

**Affiliations:** 1 Department of Anaesthesiology, Intensive Care Medicine and Pain Therapy, University Hospital Frankfurt, Goethe University Frankfurt, Frankfurt, Germany; 2 Division of Cardiology, Department of Internal Medicine III, University Hospital Frankfurt, Goethe University Frankfurt, Frankfurt, Germany

**Keywords:** Myocarditis, SARS-COV-2, Extracorporeal membrane oxygenation, Critical care, Heart failure, Impella

## Abstract

**Background:**

Primary viral myocarditis associated with severe acute respiratory syndrome coronavirus 2 (SARS-Cov2) infection is a rare diagnosis.

**Case presentation:**

We report the case of an unvaccinated, healthy patient with cardiogenic shock in the context of a COVID-19-associated myocarditis and therapy with simultaneous veno-arterial extracorporeal membrane oxygenation (VA-ECMO) and percutaneous left ventricular decompression therapy with an Impella. The aim of this review is to provide an overview of therapeutic options for patients with COVID-19-associated myocarditis.

**Conclusions:**

The majority of patients required a combination of two assist devices to achieve sufficient cardiac output until recovery of left ventricular ejection fraction. Due to the rapid onset of this fulminant cardiogenic shock immediate invasive bridging therapy in a specialized center was lifesaving.

## Background

COVID-19-associated myocarditis in non-mRNA-vaccinated patients without cardiovascular comorbidities has been reported recently [1]. However, the majority of described cases of myocarditis have been associated with mRNA (messenger ribonucleic acid) vaccinations in a younger population [2, 3].

A meta-analysis demonstrated myocardial impairment as measured by troponin I in up to 22% of critically ill COVID-19 (coronavirus disease) patients [4]. A study by Guarracino et al. gives an overview of cardiac involvement in critical COVID-19 patients based on echocardiography results [5]. Nevertheless, primary viral myocarditis associated with severe acute respiratory syndrome coronavirus 2 (SARS-CoV-2) infection is a very rare, albeit a life-threatening, disease. While the cause of myocarditis associated with mRNA vaccination remains unclear, it is well known that several viral diseases can cause severe acute myocarditis. This was also observed for SARS-CoV-2. However, cardiac failure primary due to virus-associated myocarditis under COVID-19 infection must be distinguished from other causes of cardiac failure under COVID-19 infection [6].

COVID-19-associated myocarditis has been described several times and repeatedly led to fulminant biventricular failure [7, 8]. Cardiorespiratory assist devices such as veno-arterial extracorporeal membrane oxygenation (VA-ECMO) are frequently used in patients with cardiac failure under COVID-19 infection. These are mostly patients that require the use of cardiac assist devices due to complicating secondary organ failure (e.g., as a result of obstructive shock in pulmonary artery embolism) with pre-existing COVID-19 infection. However, we will report a case of primary COVID-19-associated myocarditis embedded in our literature review.

Due to the diverse causes of acute heart failure in critically ill COVID-19 patients other than myocarditis an appropriate distinction appears to be essential [9]. Unlike other causes, covid-19-associated myocarditis often leads to rapidly progressive cardiogenic shock. Due to severe biventricular heart failure which requires sophisticated use of multiple extracorporeal devices in a specialized center [10]. With our case report and a literature review, we aim to provide insight into the different treatment options and the available literature.

## Case presentation

### Case report

With the written consent of the patient we summarised the case data from the hospital’s internal documentation. The study and all methods were performed in accordance with the guidelines of the Declaration of Helsinki. We conducted our literature search for case reports and case series according to the PRISMA Guideline. This case report was conducted according to the CARE Guideline.

#### Clinical manifestation

A 38-year-old female patient (170 cm, 65 kg, body mass index (BMI) 22.5), without pre-existing comorbidities or a family history for cardiac pathologies, presented to the emergency department with cold-like symptoms and relapsing syncope. The unvaccinated patient had been diagnosed with SARS CoV-2 infection 5 days prior to admission.

**Fig. 1:**
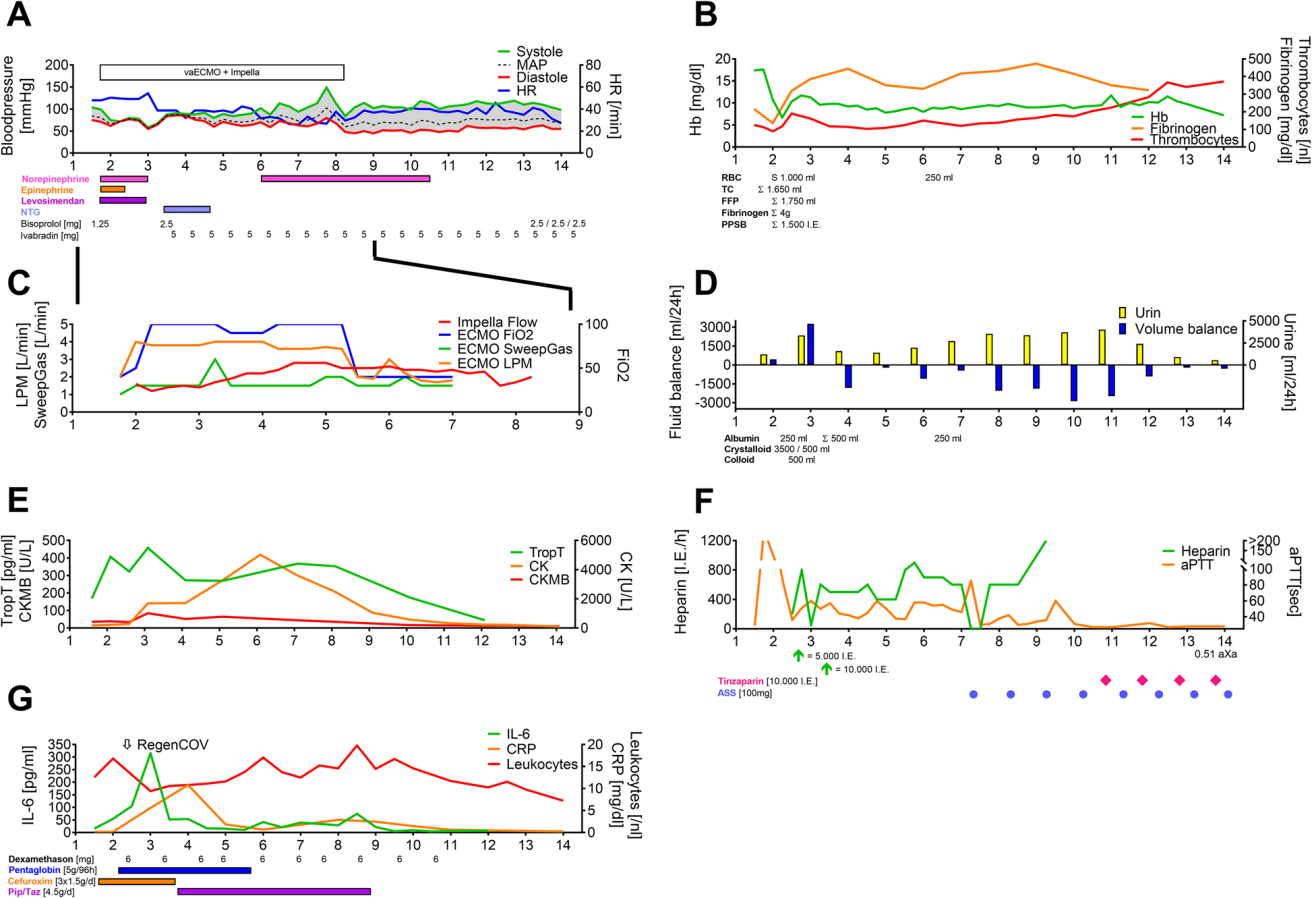
Clinical and laboratory findings Figure 1 depicts laboratory results, dosing, measurement results and ECMO settings. Abbreviations: MAP, mean arterial pressure; HR, heart rate; IVS, interventricular septum thickness; NTG, nitroglycerine; LPM, liter per minute; FiO2, inspirational oxygen concentration; TropT, troponin T; CKMB, Creatine kinase myocardial band; CK, Creatin kinase; pip/Taz, piperacillin tazobactam; Hb, Haemoglobin; RBC, red blood count; TC, thrombocyte count; FFP, fresh frozen plasma; PPSB, prothrombin complex concentrate; ASS, aspirin; aXa, anti-Xa activity; aPTT, activated partial thromboplastin time; IL-6, interleukin 6; CRP, C-reactive protein

Laboratory results showed an increased troponin T (140 pg/ml [reference range: < 14 pg/ml]; Fig. 1E) and a transthoracic echocardiography (TTE) revealed a severely impaired cardiac function. Electrocardiography (ECG) showed no signs of acute coronary syndrome. The SARS-CoV-2 RNA E-Gen cycling time was measured with 20.7 on day 2. Due to these clinical, laboratory and sonographic findings the suspected diagnosis of a COVID-19 associated myocarditis was uttered.

**Fig. 2:**
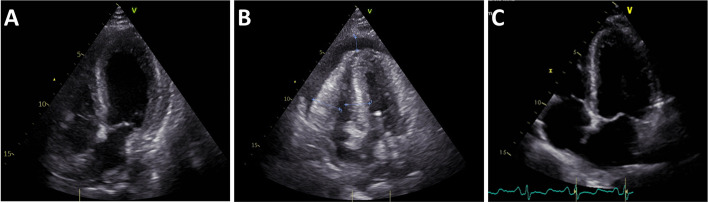
Transthoracic echocardiography. **A** Day 1. Severely impaired cardiac function, left ventricular ejection fraction ~ 20%, interventricular septum thickness (IVS) 14 mm, circular pericardial effusion (< 8 mm end-diastolic). **B** Day 2. IVS 21 mm, lateral wall thickness 25 mm, pericardial effusion (11 mm). **C** Day 15. Normalized cardiac function, left ventricular ejection fraction 65%, IVS 8 mm, no pericardial effusion.

On admission, the patient was awake, fully orientated, in a reduced general condition, cold sweating, with sinus tachycardia (122 /min) and without oxygenation disorder. TTE showed a severely impaired left ventricular ejection fraction (LVEF ~ 20%), signs of concentric left ventricular hypertrophy (interventricular septum thickness (IVS) 14 mm), a moderately reduced right ventricular function (TDI PW-doppler s-wave < 9.5 cm/s) and circular pericardial effusion (< 8 mm end-diastolic) (Fig. 2A). The patient swiftly deteriorated into cardiogenic shock with lactatemia (6.9 mmol/L) and severely impaired central venous oxygen saturation (ScvO_2_ 36%). Lactatemia quickly disappeared under sufficient circulatory support. Transpulmonary thermodilution revealed a cardiac index < 1 L/min/m^2^.

#### Drug and assist device Inotropy support

Inotropic support by means of epinephrine was initiated and awake femoro-femoral veno-arterial ECMO was provided (Cardiohelp; Getinge AB, Göteborg, Sweden) (Fig. 1 A+C). After initiation, aortic valve opening decreased severely. High dose vasopressors were needed to provide sufficient perfusion pressure (Fig. 1A). Due to reduced vigilance and ongoing hemodynamic instability the patient was intubated and ventilated (Fig. 1 A+C). In order to decompress the left ventricle (lv) and allow lv-unloading an Impella CP (Cardiac Power) device (ABIOMED; Danvers, Massachusetts, USA) was implemented (known as ECMELLA). Thereafter, hemodynamics initially stabilised. However, soon after re-admission from the heart catheter laboratory severe bleeding from the Impella cannulation site evolved, necessitating for differential coagulation analysis (thrombelastometry, impedance aggregometry) and differential therapy with colloids, crystalloids, blood products and coagulation factors (Fig. 1 B + D + F). During day 1 after ECMELLA initiation hemodynamic stabilisation could be achieved and vasopressor were reduced (Fig. 1A). Inotropic support with epinephrine was seized after ECMELLA initiation and 12.5 mg of levosimendan, was administered over a 24-hour period. Thereafter, tachycardia was treated with ivabradine 5 mg twice daily (Fig. 1A).

Cardiac function deteriorated with an increase in echocardiographic hypertrophy, most likely attributed to progressive myocardial edema (IVS 21 mm), an increase in pericardial effusion (11 mm) and a decrease in left and right ventricular contractility (Fig. 2B).

Increasing pleural effusions due to heart failure were drained. Pulmonary function remained unaffected with no clinical or radiological signs of SARS-CoV2-associated pneumonia. Figure 1 D depicts volume management, volume balance and urine production.

On day 6, a significant improvement in cardiac function, a decrease in myocardial edema and pericardial effusion was detected (Fig. 1 A+C). After gradual weaning of the extracorporeal support, the VA-ECMO was explanted on day 7 and the Impella on day 8 (Fig. 1 A+C). Based on the pulmonary gas exchange (Horovitz index: 460) the patient was extubated.

The patient recovered quickly, was transferred to the intermediate care unit on day 12 and finally discharged home on day 15. Transthoracic echocardiogram performed on discharge revealed a normalized cardiac function with no sign of persisting heart failure (Fig. 2C). ECG showed discordant terminal negative T-waves in leads V4–6 on day 13 with no change in echocardiographic function.

With appropriate rehabilitation the patient was able to fully reintegrate into her previous life.

#### Antiviral, antibacterial and immunomodulatory therapy

With stationary admission, the signs of inflammation were unremarkable (C-reactive protein 0.11 mg/dl, Interleukin-6 15.4 pg/ml and Procalcitonin 0.06 ng/ml; see Fig. 1G). The patient received dexamethasone as an immunomodulatory therapy in accordance with current guidelines (Fig. 1G) [11]. In addition, the patient received intravenous immunoglobuline (Pentaglobin, Biotest, Dreieich, Germany) 75 g over a period of 75 hours (Fig. 1G).

It is assumed that direct viral damage is the cause of COVID-19 associated myocarditis [12]. Therapy with SARS-CoV-2 specific monoclonal antibodies (1200 mg casivirimab/1200 mg imdevimab, Roche Pharma AG, Grenzach-Wyhlen, Germany) was initiated on day 2.

Serologic and PCR-testing showed no sign of viral or parasitic infection other than SARS-CoV-2. Microbial testing showed no sign of bacterial infection throughout the complete clinical course.

A myocardial biopsy was not performed because of proven suspected diagnosis, good response of therapy and the increased risk of bleeding under therapeutic anticoagulation.

## Discussion and conclusions

### Review of the literature

In order to evaluate the case, we conducted a search of the literature available.

We detected 13 cases of COVID-19 myocarditis requiring invasive cardiac organ support (Table 1) [13–24].

**Table 1:** Review to the literature

PMID/ DOI	Journal	Main author	Age	Sex	Comorbidities	Pulmonary manifestation	Time to onset^a^	Echoardiography	Coronary angiography
32275347	Eur J Heart Fail. 2020	Tavazzi et al.	69	m	None reported	Diffuse bilateral interstitial inflammation, mechanical ventilation, severe hypoxemia (requiring VAV-ECMO)	4 days	LVEF 25% Dilated LV (LV end-diastolic diameter 56 mm)	Unremarkable findings
32713771	JACC Cardiovasc Imaging. 2020	Salamanca et al.	44	m	None	Severe dyspnea, bilateral pneumonia, mechanical ventilation	~ 7 days	LVEF 15% Nondilated	Unremarkable findings
32802614	Cureus. 2020	Richard et al.	28	f	Diabetes type 1, diabetic gastroparesis, asthma, anxiety, depression	ARDS, mechanical ventilation	Recent	LVEF 26–30%	Unremarkable findings
32959998	ESC Heart Fail. 2020	Jacobs et al.	48	m	Hypertension, overweight	Cough, dyspnoea, on admission oxygen saturation 87% under room air, hypoxaemia, mechanical ventilation, prone position, ARDS, CT scan: typial pulmonary infiltrates	12 days	Hyperdynamic ventricular function (under inotropic agents and vasopressors) LV end-diastolic diameter 48 mm Interventricular septum dimension 12 mm Posterior wall dimension 11 mm	/
32997947	Circulation. 2020	Albert et al.	49	m	None	Dyspnea, on admission oxygen saturation 89% on 6 L O2 (nasal cannula) CT scan: normal lung parenchyma	14 days	LVEF 20% LV end-diastolic diamter 58 mm interventricular septum dimension 17 mm posterior wall dimension 14 mm	/
33392658	Int J Legal Med. 2021	Gauchotte et al.	69	m	Diabetes, hypertension, ischemic heart disease without chronic heart failure	None	7 days	LVEF 30% Nondilated	Non-significant lesions and two previously implanted permeable stents
33181855	Swiss Med Wkly. 2020	Othenin-Girard et al.	22	m	None	No pulmonary manifestation CT scan: normal lung parenchyma	21 days	Severe biventricular dysfunction No pericardial effusion	Aneurysm of the proximal LAD
35012323	Circulation: Heart Failure. 2022	Verma et al.	48	f	None	Shortness of breath, oxygen saturation 98% on room air Unremarkable chest imaging	5 days	LVEF 15% Thickened ventricular walls Small pericardial effusion	Unremarkable findings
32277408	Infection. 2020	Zeng et al.	63	m	None	Shortness of breath, SaO2 91.8% Typical ground-glass changes, ARDS	SARS-CoV-2 CT 22	LVEF 32% Enlarged left ventricle (61 mm) No pericardial effusion	/
33594347	Eur Heart J Case Rep. 2020	Papageorgiou et al.	43	m	Mixed Connective Tissue Disease	Cough CT scan: normal lung parenchyma	4 days	LVEF 10–15% Pericardial effusion (5 mm)	/
34125938	Kardiol Pol. 2021	Marcinkiewicz et al.	20	m	None	None	8 weeks	LVEF 15%	/
10.1097/01.ccm.0000806772.84443.86	Critical Care Medicine. 2022	Kim et al. (Case 1)	23	f	None	None	4 days	LVEF 20%	/
10.1097/01.ccm.0000806772.84443.86	Critical Care Medicine. 2022	Kim et al. (Case 2)	22	m	None	Dyspnea	3 days	LVEF 20–25%	/

MRI	Biopsy	Heart rythm abnormalities	Extracorporal device used	Antiviral, antibacterial and immunomodulatory therapy	Extracorporal device explantation alive	Duration extracorporal device (days)	Cardiac recovery (non, partial, fully)	Survival / Discharge
/	Interstitial inflammation Viral particles in interstitial cells	None	IABP VA-ECMO VAV-ECMO	None	yes	5	fully	No
Diffuse edema	No significant inflammatory infiltrates	3rd-degree AV block	temporary pacemaker VA-ECMO IABP	Methylprednisolone Tocilizumab Hydroxychloroquine Azithromycin Lopinavir-ritonavir	yes	6	fully	Yes
Myocardial necrosis, fibrosis, hyperemia	None	Episode of ventricular tachycardia, ST segment depression (lateral leads), ST elevation (I and aVL) RBBB	Impella CP	Methylprednisolone	yes	3	fully	Not reported
/	Autopsy: inflammatory infiltrates, necrosis	None on admisssion, day 5: QRS widening and a positive deflection at the end of the T wave (no hypokalaemia)	VA-ECMO	Hydroxychloroquine Azithromycin Broad-spectrum antibiotics Hydrocortisone	No	3	No	No
/	Inflammatory infiltrates, viral particles	None	VA-ECMO Impella CP	Tocilizumab Methylprednisolone Intravenous immunoglobulin	Yes	5	fully	Yes
/	Autopsy: inflammatory infiltration, SARS-CoV-2 RNA and antibodies	None	VA-ECMO	None	No	4	No	No
/	/	3rd-degree AV block, transient ST segment elevation (anterolateral leads)	Temporary pacemaker VA-ECMO	Intravenous immunoglobulins Tocilizumab	Yes	6	Not reported	Yes
/	Cardiomyocyte damage, macrophage infiltrate, SARS-CoV-2 virus	not reported	Impella CP VA-ECMO	Methylprednisolone Convalescent plasma Remdesivir Empaglifozin Tocilizumab	Yes	14	Fully	Yes
/	/	None	VA-ECMO	lLpinavir–ritonavir Interferon α-1b Methylprednisolone Immunoglobulin Piperacillin–tazobactam	No	22	Fully	No
/	No virus in cardiomyocytes, no histopathological signs of myocarditis	diffuse ST elevation and low voltage as signs of myocardial oedema	Impella CP VA-ECMO	Cefotaxime and colchicine Hydrocortisone	Yes	Impella-CP: 6 VA-ECMO: 5	Fully	Yes
Diffuse fibrosis	/	Not reported	IABP VA-ECMO	None	Yes	IABP: 6 VA-ECMO: 5	Fully	Yes
/	/	Not reported	IABP VA-ECMO	Methylprednisolone	Yes	10	Fully	Yes
/	/	Not reported	IABP VA-ECMO	Methylprednisolone	Yes	7	Fully	Yes

Presentation of the cases found within the systemic search in chart form. Report of the demographics and display of the symptoms as well as treatment parameters and outcome

^a^Time to onset Time between first symptoms and cardiogenic shock with device therapy.

*Abbreviations*: MRI Magnetic resonance imaging, VAV-ECMO Veno-arterial-venous extracorporeal membrane oxygenation, LVEF Left ventricular ejection fraction, LV Left ventricle, IABP Intra-aortic balloon pump, VA-ECMO Veno-arterial extracorporeal membrane oxygenation, AV block Atrioventricular block, RBBB Right bundle branch block, Impella CP Impella cardiac power, ARDS Acute respiratory distress syndrome, CT Computed tomography scan, SARS-CoV-2 Severe acute respiratory syndrome coronavirus 2, RNA Ribonucleic acid, LAD Left anterior descending artery

The literature search was performed (01.05.2022) by searching the MEDLINE, EMBASE, and PubMed databases using the search terms: “Myocarditis” AND “COVID-19” AND/OR “Device” AND/OR “ECMO” AND/OR “Impella” AND/OR “IABP” (intra-aortic balloon pump) were included. In a subsequent study selection we considered all case series and case reports that reported primary COVID-19 myocarditis. Case series and case reports that included myocarditis associated with mRNA vaccination against the novel COVID-19 virus were excluded.

In a secondary analysis only cases reporting severe events requiring cardiac replacement therapy were included.

### Inclusion criteria

Mechanical heart support due to presumed severe COVID-19 associated myocarditis.

### Exclusion criteria

1. Myocarditis from a cause other than SARS-CoV-2 infection.
2. Solely pharmacologic cardiogenic support
3. Association with SARS-CoV-2 vaccination

**Fig. 3:**
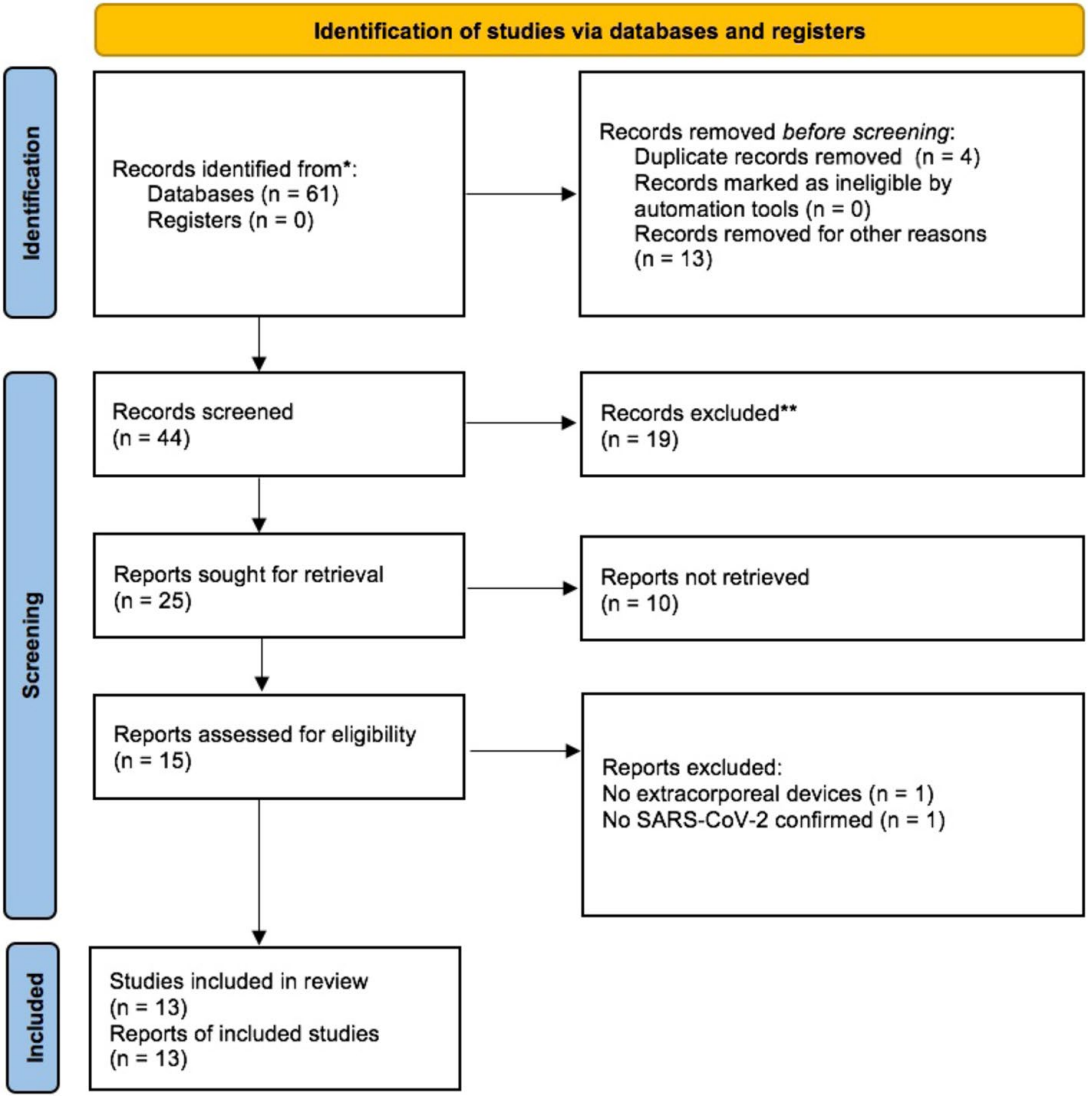
PRISMA flow diagram. Graphical representation of the systematic literature review according to the PRISMA reporting guideline. Abbreviations: SARS-CoV-2, severe acute respiratory syndrome coronavirus 2. Most of the patients in the case studies were male (m 10, f 3) and the median age was 42.6 years (interquartile range 22.5–56 years). Only three patients had reported cardiovascular risk factors diabetes and hypertension in their medical history. One of them already had two previously implanted permeable stents and an ischemic heart disease without chronic heart failure before COVID-19 myocarditis. Pulmonary manifestation in the form of pneumonia was only reported in one patient and acute respiratory distress syndrome (ARDS) was only reported in four patients of 13 cases [13–16, 21]. One of these four patients with ARDS requiring veno-arterio-venous ECMO (VAV-ECMO) due to ARDS [13].

The PRISMA flow diagram is shown in Fig. 3.

The time between first symptoms and cardiogenic shock with device implantation varied between 4 and 14 days (median 7 days, interquartile range 4–11.75 days). Only in two patients myocarditis symptoms appeared after a prolonged period with 21 days and 8 weeks.

Echocardiography revealed severely impaired LVEF in all patients. An enlarged end-diastolic diameter was described in 3 patients and pericardial effusion was only described in 2 patients.

All patients required a cardiac assist device due to fulminant impaired left ventricular ejection fraction. 12 of 13 patients received VA-ECMO therapy with additional intra-aortic balloon pump (IABP) therapy in 5 patients and Impella-CP therapy in 3 patients. One of 13 patients only received Impella therapy without VA-ECMO support. Nine patients showed cardiac recovery with extracorporeal device explantation within 1 week (7 patients) or 2 weeks (2 patients) (median 6.5 days, interquartile range 4.5–8.5 days). Two patients also required an external pacemaker due to third degree atrioventricular block.

Four patients died in the course of the disease. Two of those showed full cardiac recovery but died due to fulminant sepsis. Both had a pulmonary manifestation in the form of ARDS. The other two patients died due to multi organ failure. One of them was diagnosed with ischemic heart disease before COVID-19 myocarditis and the other one had a pulmonary manifestation in the form of ARDS.

## Discussion

We are reporting a characteristic case of an acute COVID-19-associated myocarditis. Although this form of COVID-19 disease is rare a differentiated consideration of the treatment options seems to be of high importance due to the great potential for recovery [7, 25]. If primary COVID-19 myocarditis is the main disease entity a foudroyant course with dramatic deterioration of cardiac function should be anticipated. From the previous reports, the development of a pronounced myocardial edema with lymphocytic infiltrate is evident [26]. This phenomenon could also be shown sonographically in the case treated at our clinic. This is typically associated with a marked restriction of the left ventricular ejection fraction. As in the case we described this can lead to the need for extracorporeal cardiovascular support [12, 27]. Accordingly, early allocation to a center with an appropriate expertise in cardiac assist device (VA-ECMO, Impella, IABP) should be considered. Thus, timely intervention with one or two ventricular assist devices is possible when conservative pharmacologic therapeutic approaches fail.

In the review we analyzed that total failure of left ventricular function occurred repeatedly. With single VA-ECMO therapy, inadequate aortic valve opening or lack of left ventricular ejection was reported several times, requiring dual cardiac assist devices such as VA-ECMO with Impella or VA-ECMO with IABP for adequate ventricular unloading. In the majority of the reported cases recovery was very quick, so that the inserted devices could be explanted within 1 week and the patients could leave the hospital with an almost completely normal cardiac function in the absence of complicating circumstances.

The occurrence of additional ARDS or bacterial superinfection appears to be of particular importance for the clinical outcome.

Although direct infection of myocytes through expressed ACE-2 receptors is possible, detection of viral RNA from the myocardium is usually not successful [28]. As long as pulmonary involvement is low the majority of patients survive with excellent recovery of left ventricular ejection fraction.

## Conclusion

Refractory cardiogenic shock in patients with COVID-19-associated myocarditis is a rare condition and requires rapid invasive therapy in a specialized center.

The initially acute fulminant course of a myocarditis and the LVEF can recover within days. Therefore, rapid invasive bridging therapy may save lives in patients with a SARS-CoV-2 associated myocarditis.

## Data Availability

Data cannot be shared publicly. The dataset of the case report are not publicly available due to national data protection laws but are available upon reasonable request from the corresponding author, or via the data protection officer of the University Hospital Frankfurt. Requests regarding the data can thus be sent to the corresponding author: Florian.Raimann@kgu.de or Datenschutz@kgu.de.

## Abbreviations

ACE-2 receptors: Angiotensin Converting Enzyme 2 receptors
ARDS: Acute respiratory distress syndrome
AV block: Atrioventricular block
BMI: Body mass index
CT: Computed tomography scan
ECG: Electrocardiography
IABP: Intra-aortic balloon pump
Impella CP: Impella Cardiac Power
LAD: Left anterior descending artery
LVEF: Left ventricular ejection fraction
LV: Left ventricle
MRI: Magnetic Resonance Imaging
RBBB: Right bundle branch block
RNA: Ribonucleic acid
SARS-CoV-2: Severe acute respiratory syndrome coronavirus 2
TTE: Transthoracic echocardiography
VAV-ECMO: Veno-arterial-venous extracorporeal membrane oxygenation,
VA-ECMO: Veno-arterial extracorporeal membrane oxygenation.
